# Women in focus: advice from the front lines on how to enable well-being and build resilience

**DOI:** 10.1186/s13244-020-00858-7

**Published:** 2020-03-30

**Authors:** Elizabeth Morris, Rahel A. Kubik-Huch, May Abdel-Wahab, Elizabeth Balogun, Charlotte Beardmore, Regina Beets-Tan, Aidan Boyd-Thorpe, Lorenzo Derchi, Michael Fuchsjäger, Janet Husband, Valerie Jackson, Dina Husseiny Salama, Valérie Vilgrain, Hedvig Hricak

**Affiliations:** 1 Department of Radiology, Memorial Sloan Kettering Cancer Center, 1275 York Avenue, C-278, New York, NY 10065 USA; 2 Department of Medical Services, Institute of Radiology, Kantonsspital Baden, Baden, Switzerland; 3 Division of Human Health, International Atomic Energy Agency, Vienna, Austria; 4 National Orthopaedic Hospital, Igbobi, Lagos, Nigeria; 5 The Society and College of Radiographers, London, UK; 6 Department of Radiology, The Netherlands Cancer Institute, Amsterdam and University of Maastricht, Maastricht, The Netherlands; 7 Communications and Multimedia, European Society of Radiology, Vienna, Austria; 8 Department of Radiology, University of Genoa, Genoa, Italy; 9 Division of General Radiology, Department of Radiology, Medical University of Graz, Graz, Austria; 10 Institute of Cancer Research, London, UK; 11 American Board of Radiology, Tucson, AZ USA; 12 Department of Radiology, Egyptian Atomic Energy Authority, Cairo, Egypt; 13 APHP, HUPNVS, Hôpital Beaujon, Clichy and Université de Paris, Paris, France

**Keywords:** Leadership, Resilience, Well-being, Radiology, Gender, Diversity, Mentoring

## Abstract

The 2019 European Congress of Radiology program, “Women in Focus: Be Inspired,” offered insights from successful women and men for overcoming a number of everyday work and personal life challenges. With regard to balancing career and personal life and achieving well-being, the advice of female and male radiology leaders on the front lines, throughout the world, shares common themes. This paper highlights and expands on points of advice and encouragement from the “Women in Focus” program. The first step is to know yourself, so you can set priorities. Then, take charge, be brave, and follow your dreams, which may not be the same as other people’s. Finding balance requires examining your goals and acknowledging that you may not be able to get everything you want all at once. Receiving effective mentorship from numerous sources is key, as is finding an environment that supports your growth. It is important to surround yourself both at work and at home with people who support your ideas and give you a sense of peace, well-being, and resilience. If the culture does not fit, have the courage to move on. Current leaders should reach out to ensure the diversity of future teams. Society benefits, radiology benefits, and our patients benefit from a specialty that values equity, diversity, and inclusiveness.

## Key points

- Know yourself and choose the career path that is right for *you*.
- Do not regret your life choices, but learn from them and evolve.
- Maintaining mutually supportive relationships with family and friends is key to well-being.
- Mentorship in radiology can improve clinical skills, research output, and career satisfaction.
- Reach for the sky!

## Introduction

**Fig. 1:**
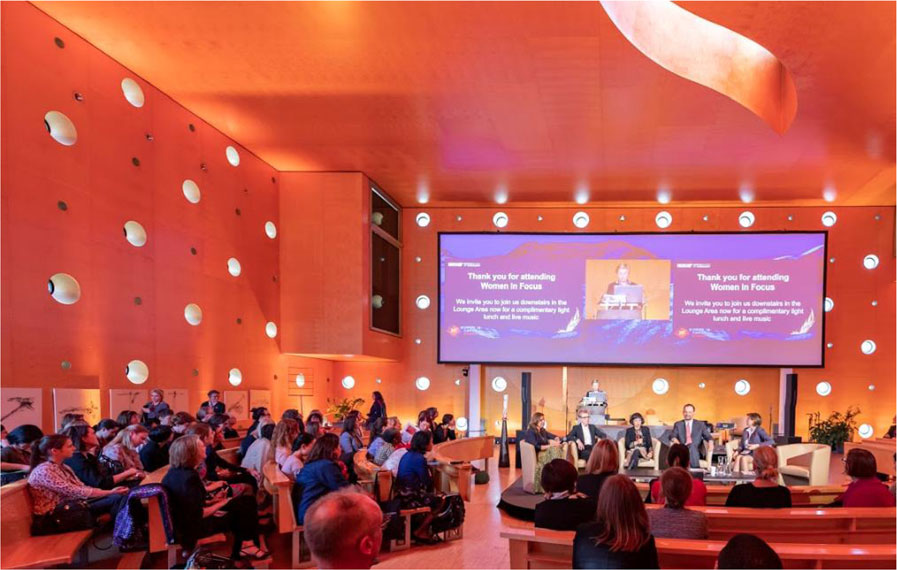
The “Women in Focus” program took place at the European Congress of Radiology 2019. Over the course of four inspiring sessions, expert speakers and panelists provided their unique insights into leadership, mentoring, life balance, and many other important topics affecting professionals working in healthcare

The 2019 European Congress of Radiology program, “Women in Focus: Be Inspired,” offered insights from successful women and men for overcoming a number of everyday work and personal life challenges (Fig. 1). While the emphasis was on the needs of women, much of the content was gender neutral and applied to all those concerned with either choosing their career path or finding balance on the path they had already chosen.

**Fig. 2:**
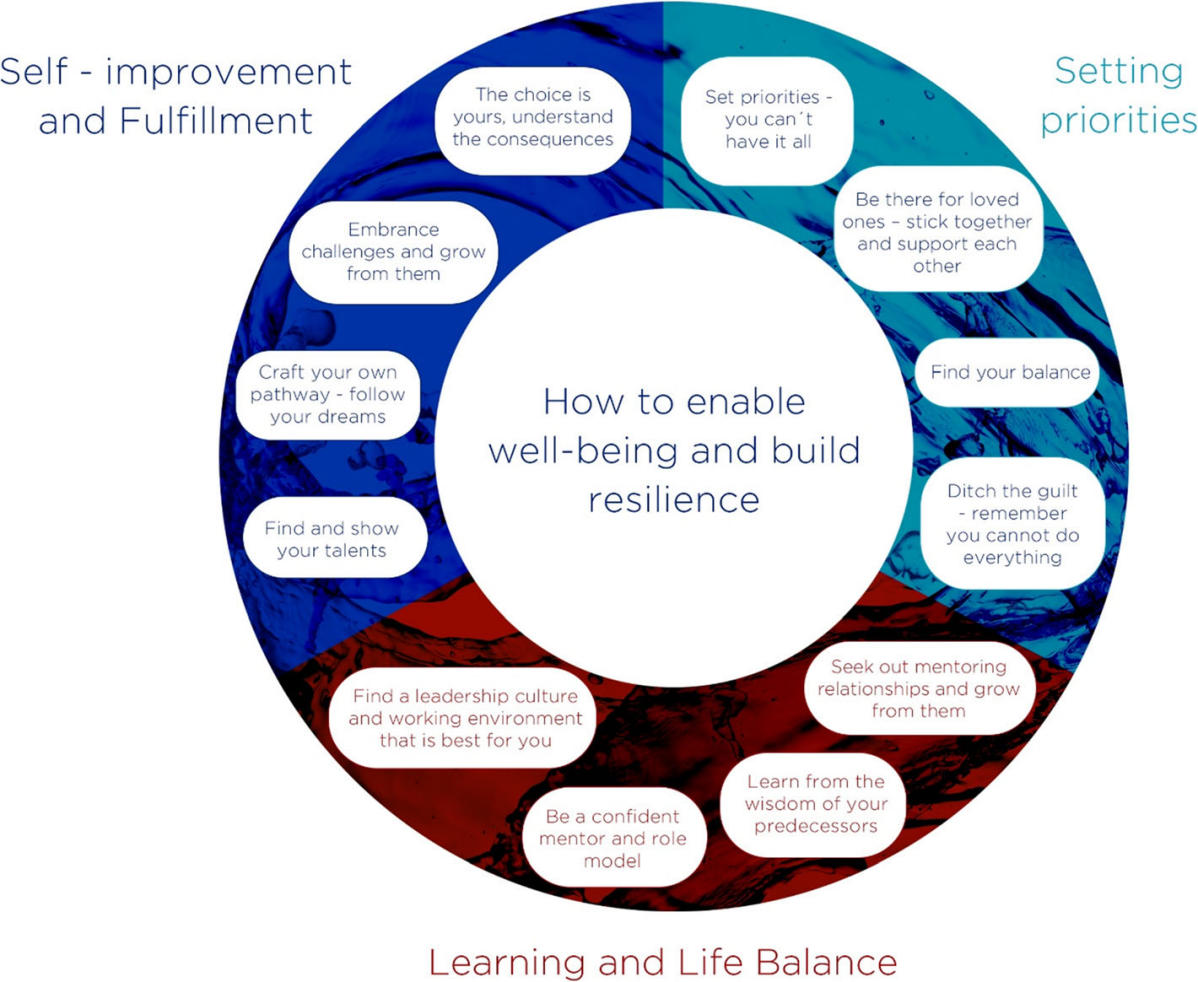
A summary of points of advice and encouragement discussed in the text on how to enable well-being and build resilience

In keeping with the relaxed and personable spirit of the event, this paper highlights and expands on points of advice and encouragement—some drawn from personal experience and some from research, some practical and some inspirational (Fig. 2). Each section begins with a quotation from one of the program’s culturally diverse array of speakers and panelists, followed by a further explanation from that individual.

We wholeheartedly believe that cultural and gender diversity in the field of radiology will benefit radiologists themselves as well as the patients they care for. We hope readers at all career stages, women and men around the world, find the material below both inspiring and useful for building the skills, resilience, and well-being needed to achieve their potential and realize their dreams.

## Setting priorities

*“The choice is yours.”*

– Hedvig Hricak

Each of us must decide: What do we value most, what do we really care about, what is the most important thing for our well-being? And then we have to choose. That is why we should take time to get to know ourselves.

Physicians are fortunate to have many options to choose from in charting their career paths. At the end of medical school, the first big choice is that of a subspecialty. Many people find out that their initial choice does not really suit them and make a switch—which is perfectly all right and even laudable. Moving forward, the next big choice is between private practice and academia. Making a switch after this decision can be somewhat more difficult, though certainly not impossible. Gaining research experience during residency is advisable not only to enhance your knowledge but also to help decide whether you like research and see it as a more satisfying career path for yourself. You need to ask yourself whether, 5, 10, or even 20 years down the road, you will still be interested in the work you have chosen. Thirdly, regardless of whether you go into academia or private practice, you may need to decide at some point whether to go part time to address family or other personal needs. This decision is not irreversible, and it is certainly possible to go part time for a period of time and then return to full-time status. However, making the transitions back and forth may prove challenging, both practically and emotionally. Every decision you make will carry consequences, and only you know what is right for you. You can seek advice, discuss options with family, friends, and mentors, but in the end, the decision has to be yours. Do not simply allow decisions to be made for you. Rather, do your best to anticipate the consequences of your choices and then accept responsibility for them, knowing that by doing so, you will be better able to look forward to your future. Above all, always remember: When selecting a career path, there is no right or wrong. What matters is what is right for *you*. Regardless of what you choose, never, ever stop learning. That is important not only to keep improving patient care, but because improving yourself enhances personal satisfaction.

*“You can’t have it all – it is all about the right choices.”*

*–* Rahel Kubik-Huch

Many careers are “nonlinear,” meaning they have not followed a conventional trajectory [1]. As you carve out your career path, there will likely be times when you have to set priorities, choosing between work goals and the concerns of personal life. For instance, if you are in a dual career couple, you and your partner will likely decide to coordinate your careers with regard to your place of residence. In the case of marriage, especially for women, if you decide to accept your new spouse’s name, you will have to consider that it may have an impact on your visibility as an academician (use an ORCID ID for your publications to help ensure that your work continues to be recognized [see www.orcid.org]!). Moreover, particularly for women of reproductive age, it may become necessary to decide whether to fully focus on a professional career, to focus on family life with kids, or to try to achieve a balance of both. While the division of labor at home is becoming more equal than it was in the past, women with children often still take the larger share of responsibility for family management and chose to work part time [2].

You cannot have it all—or at least, you cannot always have it all at the same time. Working part time for a limited period or even taking a break for family or other reasons is not necessarily a decision against a career. According to the Harvard Business Review, 36% of highly qualified female professionals said they had worked part time for some period of time to balance work and personal life [1]. Nevertheless, it is important to remain involved in your field of specialization, to maintain your professional network and always be reliable and meet deadlines. Nobody cares what your excuse is if you do not deliver; only the results count.

Never look back, never regret: In retrospect, some of your life choices may seem like mistakes, but they might have felt right when you made them. Even if not, do not regret these choices, but learn from them and evolve.

*“Set your priorities and always remember to be there for your loved ones.”*

– Michael Fuchsjäger

Pursuing a successful career when you are married and have a family may require a lot of careful balancing of your professional and personal lives. You should, of course, set priorities, but sticking to them in reality might not prove easy. It definitely helps to never touch holiday seasons or family vacations, which usually follow your children’s schedules. No matter how successful you are, no matter how important your attendance at a meeting might be, you have to be available when your family needs you, even if this means altering your plans. In the end, you will not be remembered for giving one more lecture or publishing one more paper, but by your emotional impact and the personal imprint you leave as a human being.

Building trust and communication is highly important in every professional relationship [3]. It is even more important in your relationships with your partner and family. You and your partner should enjoy each other’s successes and share any unhappiness over setbacks. This will not only help you in your professional careers but will bring you closer. Educating each other about your jobs may even be fun and will increase mutual respect. Keep in mind that you must never, ever disparage your partner’s occupation.

Relocating to advance your career can sometimes be very tempting. Based on my experience, I believe you should always try to relocate together or not let any separation last long. I have had the good fortune to work internationally, and the decision to accept international professional opportunities was always shared together with my spouse. I cannot imagine how we could have proceeded differently. The company and support of my spouse and children provide an incredible feeling of security as well as a constant source of energy and resilience.

## Women in focus

*“Our gender is not a choice, but therein lies our strength.”*

– Elizabeth Balogun

The effects of gender-based stereotypes differ between cultures as well as specific work environments [4–6]. Here, I would like to address the concerns of women in challenging environments where gender stereotypes pose major barriers to the advancement of women in the workplace.

There are still many places in the world where a woman is expected to be seen and not heard. It is challenging for women to not have a voice. Even if their voice is heard, women may not be placed in certain work roles, because it is presumed that they will likely marry and relocate or start a family that will demand much of their time and will therefore diminish their professional commitment to their employer. Denials of work opportunities based on such stereotypes have had long-term effects on the lives of countless women, causing them to feel frustrated and underappreciated and to lament that their capabilities have been shortchanged. By giving a voice to women and recognizing that these stereotypes hold women back, we can bring resilience to those who seek out opportunities.

It often requires a huge amount of personal strength for a woman to pursue a desire for professional growth in these challenging environments. Therefore, it is important to look beyond the barriers associated with traditional feminine roles and focus on the strengths associated with them instead. Traits commonly considered feminine and associated with the maternal role include the ability to nurture, flexibility, resilience, good listening skills, and the capacity to be a loyal “team player” who consistently puts collective interests ahead of personal concerns. Women with these strengths should view them as a reason to hold their heads high in professional roles and to feel confident about serving as mentors and role models. Additionally, adopting methods to build resilience and well-being can further empower women in their search for opportunity.

*“Women are not inferior or superior to men—your achievements will tell.”*

*–* Dina Husseiny Salama

In addressing gender disparities in the workplace, we should strive to deliver equal career opportunities for women and men, so that women can fully contribute and be rewarded for whatever abilities they have to offer. For a very limited number of positions, gender preferences in hiring may be valid due to physiological discrepancies between women and men. However, in general, our focus should be on abolishing any discrimination against women, as progress in this effort will benefit not only women but society as a whole.

Empowering women is preferable to mandating their appointment to certain positions. It is better to ensure that all job opportunities are accessible to women who are qualified for them than to assign an obligatory quota of women for each job. There is no doubt that many people still have conscious or unconscious biases against women. I have found in my experience that some of the greatest barriers to women ascending to and flourishing in careers have often come from other women. Just because we are women does not mean we are not influenced by the biases that are found in our societies.

If we want to succeed in eliminating gender discrimination, it is essential for women to look within themselves, evaluate their own biases, and strive to be aware of their own attitudes. As women, we should make a conscious effort to avoid creating unnecessary obstacles to other women and we should celebrate them when they achieve. This will help to accelerate progress, as the more often women succeed in leadership positions, the greater general social acceptance of women in leadership will become.

*“Gender diversity in our profession is a step forward not only for radiologists, but also for patients.”*

– Lorenzo Derchi

The gender gap between women and men in medicine is closing. Women constitute more than half of students in medical schools almost everywhere, and their representation in radiology is also growing, at least in Europe: A 2018 survey showed that at 48% of European departments of radiology, 25 to 50% of radiologists were women, and at about 42%, the majority were women [7, 8].

By contrast, the gender gap remains wide in leadership positions in medicine and in radiology specifically. For example, data published in 2014 indicated that at American medical schools, women represented only 21% of full professors and 16% of deans [9], and currently, among the 428 department heads in the European Society of Radiology database, only 20.6% are women. As academic success tends to be linked to advancement to leadership positions, closing this gap will require, among other steps, consciously encouraging women’s participation in research, which already lags behind men’s at the residency stage [10].

Will balancing the proportions of male and female radiologists bring beneficial changes to the clinical practice of radiology departments? Surely, the answer is yes. In any sector, diversity in the workforce is considered a strength that helps organizations provide better services to their diverse clientele. In medicine, it is useful to have a workforce that mirrors the patient population. For instance, women patients often prefer female doctors [11].

Diversity in leadership has been linked with a host of tangible benefits in the business sector. Achieving gender balance in leadership positions will surely improve how radiology departments and academic institutions perform, not only in business terms but also with respect to the service given to patients.

To do the best we possibly can for all patients, male and female, and to create work environments equally favorable to the well-being of healthcare practitioners of both genders, we should strive for gender balance throughout medicine, including in radiology.

## Learning and life balance

*“Show your talents and find your balance.”*

– Valérie Vilgrain

We are all talented but in many different ways. The statement “show your talents” could appear pretentious, but the idea behind it is to get to know yourself better. In a highly connected environment, where our best friend is our cell phone, we often fail to look within ourselves. I would certainly have saved a lot of time and avoided many mistakes if I had worked harder on knowing myself before reaching the age of maturity! Better knowing and showing our talents helps us to build them into skills and abilities. Yet, it is equally important to recognize what we are bad at and to make appropriate choices.

Is it easy to scrutinize ourselves? Certainly not, and it may take time, but it improves our ability to communicate in professional and personal life. We have all experienced fantastic times, with great work-life balance, as well as tough times. Balance is like a cooking recipe, very personal. We get the standard ingredients, but we adapt the recipe with a personal touch, which makes the dish very special. My personal one is as follows: First I manage my life and not the lives of others! Second, what is important for me (family, friends, hobbies…) is a priority, and I protect it.

Speak up and be confident!

*“Effective mentorship is essential to reach your full potential.”*

– May Abdel-Wahab

Mentorship is needed throughout our professional lives to support career development, work/life balance decisions, and navigation of the political landscape. Since needs and situations change, one should constantly seek out new mentors. Mentorship in medicine can lead to improved clinical care as well as greater research output and career satisfaction [12, 13].

Given the persistence of gender disparities in leadership roles in medicine, including radiology, there is a scarcity of motivating female role models and mentors, which in itself represents a barrier to the academic advancement of women [14]. In addition, there is a need to create substitute networking experiences for women outside the traditional “old boys’” networks [2].

In some places, mentoring programs (or broader programs that include improved access to mentoring) have been developed to help women achieve success in academic medicine and assume leadership positions [14, 15]. Careful pairing of mentor to mentee is essential, and appropriate pairing is more likely when the mentor is selected by the mentee [16]. Once an effective mentorship relationship is established, sustainability is important. New demands on time or resources experienced by either party, or negative behaviors displayed in the relationship, can lead to a decrease in the quality or even cessation of mentorship. One must remember that both mentor and mentee contribute to the effectiveness or dysfunction of the relationship. A trusting, authentic, engaged, caring, bi-directional relationship is most likely to be effective [17].

There are ample opportunities for mentoring relationships to be established, both informally and through formal programs, including international programs. Let us remember to utilize all the means we have at our disposal to create fruitful, rewarding mentoring relationships today.

*“Differences in leadership are more generational than gender driven.”*

– Elizabeth Morris

We have been taught to believe from a young age that girls and boys are different. Translating this belief to the workplace, we may assume that women and men have fundamentally different values and styles. Traditional conceptions of the roles of men and women in the workplace reinforce this assumption [2, 18, 19]. Similarly, we tend to make assumptions about different generations. For example, baby boomers are expected to be driven and hardworking, while millennials are expected to be idealistic and embrace technology [2, 18, 20].

Traditional expectations about approaches to leadership based on gender alone are less relevant to a generation that embraces greater equity, diversity, and inclusion in the workplace. Younger people, who have lived through an era of accelerated change, have generally developed greater tolerance for the diversity in the world, a greater desire for diversity in the workplace, and more openness to different styles of leadership. Interestingly, compared to baby boomers, younger people—especially younger men—place a higher value on humility in leaders [18]. Furthermore, the proportions of men (31%) and women (27%) aiming to reach the “c-suite” are now roughly even [18].

Regardless of gender, the desire to push limits, having the courage to fearlessly jump in, will allow you to grow, thrive, and take on new opportunities. Cultures vary between institutions and are affected by the gender and generational diversity of the team. Seek an institution in which the leadership culture is compatible with your needs and expectations. Resilience is only possible in an environment where you feel safe, protected, and unafraid to use your voice.

*“In a lifetime, you will have many mentors for many reasons—your inbox will never be empty.”*

– Valerie Jackson

Many people feel that mentoring must be a formal relationship. In my experience, the best mentors have been unplanned and unassigned. While the relationships have been informal, these individuals have had a huge impact on my life and career. The key is to be open to, or actively seek, the support, guidance, and wisdom of others. Early in my training and career, I was mentored by my department chair and the chief of radiology at the hospital where I practiced. In addition, I benefited from peer-to-peer mentorship; there were several of us who were new on the faculty who shared whatever meager expertise we had with each other. Over the years, various individuals provided informal mentorship as I became involved in service to professional organizations in radiology. Do not discount the value of mentors for your personal life. This is especially important for women, where the seemingly overwhelming number of tasks associated with caring for children and/or elders and running a household can make professional success more difficult.

Many of us are driven overachievers. Even those of us who procrastinate want to feel the satisfaction of completing our tasks. In today’s world of ever-increasing workloads and speed of communication, it is difficult to prioritize and nearly impossible to keep up. Even if all “tasks,” such as emails or cases on the PACS worklist, are done, within seconds, there are more. This is especially true for people in leadership positions, because we have many people depending on our quick but thoughtful decisions. Early in my tenure as a radiology department chair, I was lamenting to my boss, the Dean of the medical school, that I felt I was never caught up. He said, “Your inbox will never be empty. Don’t waste your time and energy feeling guilty about it. Learn to prioritize to the best of your ability and remember that you can’t do everything.” This advice has served me well over the years. I have learned to ditch the guilt (well, at least some of it), delegate things that someone else can do, and ignore when appropriate. That leaves me time to prioritize activities and focus my attention on the most important tasks in a timely manner. Am I perfect at this? No, but I find my life is now much less stressful.

*“If I have seen a little further, it is by standing on the shoulders of giants.”*

– Regina Beets-Tan

This quote from Sir Isaac Newton dates to 1675 and still holds true today, because the opportunities we have to develop and progress in our chosen careers often spring from the achievements and vision of our predecessors. If we are receptive to their wisdom, we cannot only master it but move beyond it. In essence, the greatness of those who went before us lifts us up, allowing us to explore untrodden paths and reach new horizons. This metaphor has always been central in my career. Over the years, great mentors have helped me overcome obstacles and recognize opportunities to advance. Now, having the privilege of mentoring my junior faculty, I have learned that it also works the other way around. For me, mentoring is an extremely positive experience, and in passing on my own wisdom, I benefit in return. Not only do I enjoy the sessions with my mentees, I also acquire more knowledge. Furthermore, their eagerness to learn, their positive attitudes, and their ambition to reach their goals keep me inspired. Nothing makes me prouder or happier than seeing my mentees realize their potential and grow into great leaders.

## Self-improvement and fulfilment

*“Be bold and grasp the opportunities – push open the doors.”*

– Charlotte Beardmore

The recipe for success has “just four essential ingredients,” stated the late Benjamin Franklin Fairless, a former Chairman of the United States Steel Corporation. “Choose a career you love, give it the best there is in you, seize your opportunities, and be a member of the team.” These four essential ingredients have helped support and develop my skills and given me confidence to seize opportunities throughout my career [21].

Working with great professionals, who have been full of enthusiasm, drive, and commitment and have shined new light into my world, has helped provide me with the vision and confidence to identify and pursue key opportunities. My role models have been important to me, leading the way often in small steps and offering valuable guidance.

Juggling my professional career with a family required me to be flexible, resilient, and open-minded. Completing an MBA also increased my confidence. This, coupled with my belief that we can all make a difference in the world through small actions and steps, supported me in striving to continually grow and look outwards.

So, my message is this: Each of us has an open book. Craft your own pathway, have confidence and push forward, taking the opportunities that come into your life. Each door opens upon a new world, a new garden of possibilities. There will be challenges, which in themselves will offer chances to grow in a new context.

As Tom Peters, the management guru, stated, “If a window of opportunity appears, don’t pull down the shade” [22].

*“Reach for the sky.”*

– Janet Husband

The era of sophisticated computer technology which enshrouds so many aspects of our lives today has not only revolutionized the whole practice of medicine but has also created unprecedented opportunities to develop an exciting and fulfilling career in academia. There are few specialities where this is more apt than radiology. However, such opportunities often require choices to be made where a vision of the path ahead may not be clear, and success not guaranteed. It is easy to be discouraged from such a “leap into the dark” and to feel unprepared and inadequate, but the best advice is to be brave, follow your dreams, and if you want to achieve your goal above all else, then you will succeed. The way ahead will be fraught with challenges and difficulties as well as success, so inevitably there will “ups and downs,” but a good mentor who has traveled the same road will be your best guide. Such a mentor is, I believe, critical to your success as you forge ahead and you, too, become a leader. On this journey, it is important to bring others with you and to recognize their contribution to your success, for seldom has anyone achieved a strong leadership position without the support of a loyal team. So, follow your dreams, meet your challenges, and you will be rewarded. The essence of this advice is elegantly stated in the motto of the Royal Airforce: *Per ardua ad astra*—a Latin phrase interpreted as meaning “through adversity to the stars.”

## Conclusion

With regard to balancing career and personal life and achieving well-being, the advice of female and male radiology leaders on the front lines, throughout the world, shares common themes. The first step is to know yourself, so you can set priorities. Finding balance requires personal evaluation, realizing that you may not get everything you want all at once. Take charge, be brave, and follow your dreams, which may not be the same as other people’s. Embrace every opportunity, believe in your capabilities, and participate in influential groups to achieve visibility. Receiving effective mentorship from numerous sources is key to success, as is finding an environment that supports your growth. It is important to surround yourself both at work and at home with people who support your ideas and give you a sense of peace, well-being, and resilience. If the culture does not fit, have the courage to move on. Current leaders should reach out to ensure the diversity of future teams and provide mentorship. Society benefits, radiology benefits, and our patients benefit from a specialty that values equity, diversity, and inclusiveness.

## Data Availability

Not applicable.
